# Mitochondrially targeted ceramide LCL-30 inhibits colorectal cancer in mice

**DOI:** 10.1038/sj.bjc.6604099

**Published:** 2007-11-20

**Authors:** F Dahm, A Bielawska, A Nocito, P Georgiev, Z M Szulc, J Bielawski, W Jochum, D Dindo, Y A Hannun, P-A Clavien

**Affiliations:** 1 Swiss HPB (Hepato-Pancreato-Biliary) Centre, Department of Visceral and Transplantation Surgery, University Hospital Zurich, Rämistrasse 100, Zurich CH-8091, Switzerland; 2 Department of Biochemistry and Molecular Biology, Medical University of South Carolina, 173 Ashley Avenue, Charleston, SC 29425, USA; 3 Department of Pathology, University Hospital Zurich, Rämistrasse 100, Zurich CH-8091, Switzerland

**Keywords:** colorectal neoplasms, cell death, ceramides, ceramidoids, pharmacokinetics, drug therapy

## Abstract

The sphingolipid ceramide is intimately involved in the growth, differentiation, senescence, and death of normal and cancerous cells. Mitochondria are increasingly appreciated to play a key role in ceramide-induced cell death. Recent work showed the C16-pyridinium ceramide analogue LCL-30 to induce cell death *in vitro* by mitochondrial targeting. The aim of the current study was to translate these results to an *in vivo* model. We found that LCL-30 accumulated in mitochondria in the murine colorectal cancer cell line CT-26 and reduced cellular ATP content, leading to dose- and time-dependent cytotoxicity. Although the mitochondrial levels of sphingosine-1-phosphate (S1P) became elevated, transcription levels of ceramide-metabolising enzymes were not affected. In mice, LCL-30 was rapidly absorbed from the peritoneal cavity and cleared from the circulation within 24 h, but local peritoneal toxicity was dose-limiting. In a model of subcutaneous tumour inoculation, LCL-30 significantly reduced the proliferative activity and the growth rate of established tumours. Sphingolipid profiles in tumour tissue also showed increased levels of S1P. In summary, we present the first *in vivo* application of a long-chain pyridinium ceramide for the treatment of experimental metastatic colorectal cancer, together with its pharmacokinetic parameters. LCL-30 was an efficacious and safe agent. Future studies should identify an improved application route and effective partners for combination treatment.

The last two decades have seen an explosive growth in the understanding of sphingolipid biology. Initially considered inert structural constituents of cell membranes or precursors thereof, sphingolipids have emerged as key messenger and bioactive molecules in a wide range of biological processes (Futerman and Hannun, 2004). The sphingolipid ceramide can be formed by the breakdown of sphingomyelin or through *de novo* synthesis. It is intimately involved in growth, differentiation, senescence, and death of normal and cancerous cells. Several inductors of cell death, for example, TNF*α* (Obeid *et al*, 1993), anthracyclines (Bose *et al*, 1995), or irradiation (El-Assaad *et al*, 2003) involve ceramide signalling. Administration of exogenous ceramide also causes cell death in various cancer cell lines (Oh *et al*, 1998; Bras *et al*, 2000). It is noteworthy that, many cancer cells have a specific ‘sphingolipid–phenotype’, including lower endogenous ceramide levels (Itoh *et al*, 2003) and a higher sensitivity to the effects of exogenous ceramide (Selzner *et al*, 2001). This offers the opportunity to selectively target cancer cells with ceramide compounds.

Mitochondria are increasingly appreciated to play a key role in ceramide-induced cell death. Ceramide treatment of isolated mitochondria leads to the activation of a mitochondrial protein phosphatase (PP2A), which dephosphorylates the antiapoptotic Bcl-2 (Ruvolo *et al*, 1999) and causes cytochrome *c* release (Ghafourifar *et al*, 1999). Furthermore, ceramide has been shown to inhibit mitochondrial complex I (Di Paola *et al*, 2000) and to induce the formation of reactive oxygen species in mitochondria (Garcia-Ruiz *et al*, 1997). Targeted delivery of sphingomyelinase to mitochondria, but not to other subcellular compartments, results in bax translocation and the activation of the mitochondrial pathway of apoptosis (Birbes *et al*, 2001).

The central role of mitochondria in ceramide-induced cell death makes them an alluring target for the specific delivery of ceramide compounds. Naturally occurring ceramides contain a relatively long N-linked fatty acyl chain (14–24 carbon atoms), rendering them practically insoluble in water. Ceramides modified with a *ω*-pyridinium moiety contain a positive charge delocalised over the *π*-electron system (Szulc *et al*, 2006). These ceramide analogues exhibit a much higher water solubility and preferentially accumulate within the mitochondrial matrix driven by the electrochemical gradient (Novgorodov *et al*, 2005; Dindo *et al*, 2006; Senkal *et al*, 2006). The approach of attacking mitochondria is further supported by the fact that cancer cells’ mitochondrial membrane potential tends to be more polarised than that of normal cells (Chen, 1988).

Despite many pleas for ceramide-based treatment regimens against cancer (Radin, 2003), progression from cell-culture to *in vivo* applications has been slow, and no clinical trials have been reported to date. Previous work in animal models has shown the ceramidase inhibitor B13 to profoundly suppress the growth of colorectal liver metastases (Selzner *et al*, 2001) and to reduce the progression of metastatic prostate cancer (Samsel *et al*, 2004). A short-chain pyridinium ceramide (C6-analogue) was recently shown to inhibit tumour growth in a model of metastatic head and neck squamous cell carcinoma (Senkal *et al*, 2006). These studies illustrate the large potential of a targeted approach to cancer therapy by interference with ceramide signalling.

Our recent work showed LCL-30, a C16-pyridinium ceramide analogue, to induce cell death *in vitro* by mitochondrial targeting (Dindo *et al*, 2006). The aim of the current study was to translate these results to an *in vivo* model. This study represents the first application of a long-chain pyridinium ceramide *in vivo* as well as a determination of its tolerability and efficacy in a widely used animal model of metastatic colorectal cancer.

## MATERIAL AND METHODS

### Cell culture and biological reagents

D-erythro-2-*N*-(16′-(1”-Pyridinium)-hexadecanoyl)-sphingosine bromide (LCL30) was prepared in the Lipidomics Core of the Medical University of South Carolina (Szulc *et al*, 2006). CT-26 murine colon carcinoma cells (ATCC, Manassas, VA, USA) were cultured in RPMI Medium (Invitrogen, Basel, Switzerland) supplemented with 10% fetal bovine serum (PAA Laboratories, Austria), 100 U ml^−1^ of penicillin, and 100 *μ*g ml^−1^ of streptomycin (Invitrogen). The cells were maintained at 37°C in a 5% CO_2_ atmosphere. Actinomycin D and doxorubicin hydrochloride were from Sigma-Aldrich (Buchs, Switzerland). Recombinant human TNF*α* was purchased from R&D Systems Inc. (Minneapolis, MN, USA). Caspase-3 and -8 substrates (Ac-DEVD-AFC and Ac-LETD-AFC, respectively) as well as caspase-3- and pan-caspase-inhibitor (Z-DEVD-CHO and Z-VAD-fmk, respectively) were from Alexis Biochemicals (Lausen, Switzerland).

### Cell viability assay

Cells were seeded into 12-well plates at a density of approximately 50%, corresponding to 5 × 10^5^ cells per well and allowed to adhere overnight, before the medium was changed to the specified conditions. The MTT assay was performed as described previously (Dindo *et al*, 2006). In parallel, cells were detached using 1% trypsin (Invitrogen) and centrifuged at 800 g. Cell pellets were resuspended in PBS with trypan blue (Sigma-Aldrich) and both stained and unstained cells were counted.

### Mitochondrial isolation and determination of cytochrome *c*

All procedures were performed on ice. Cells were scraped and washed twice in PBS before being resuspended in five volumes of isolation buffer (250 mM sucrose, 20 mM HEPES, pH 7.5, 10 mM KCl, 1.5 mM MgCl_2_, 1 mM EDTA, 1 mM EGTA, 1 mM DTT, 0.2 mM PMSF). Cells were broken by repeated aspiration through a pipette. Centrifugation for 10 min at 700 g yielded unbroken cells as well as nuclei. Supernatants were centrifuged for 15 min at 10 000 g to pellet a crude mitochondrial fraction. Mitochondrial enrichment was confirmed by Western blotting for cytochrome *c* oxidase complex IV (Abcam, 20E8, Cambridge, UK). Mitochondrial cytochrome *c* release into the cytosol was assessed quantitatively with the Quantikine enzyme-linked immunosorbent assay kit (R&D Systems, Abingdon, UK).

### ATP measurement

Cellular ATP content was measured in cellular lysates with the Enliten Luciferase/Luciferin reagent (Promega, Mannheim, Germany) according to the manufacturer's instructions and normalised to protein content.

### Caspase-3/-8 activities

Cells were scraped and lysed (10 mM Tris–HCl, pH 7.4, 2 mM EDTA, 0.1% NP-40) for 10 min at 4°C. After centrifugation for 10 min at 10 000 g, the lysate corresponding to 25 *μ*g of protein was incubated for 30 min at room temperature with or without 1 *μ*M caspase-3 inhibitor Z-DEVD-FMK. Then, caspase-3 substrate Ac-DEVD-AFC (10 *μ*M) or caspase-8 substrate Ac-LETD-AFC (10 *μ*M) and dithiothreitol (10 mM final concentration) were added, and enzyme activity was monitored by measuring fluorescence at 390_ex_/538_em_ nm (Biolise software and Fluostar microtiter plate reader, Crailsheim, Germany). Caspase activity was then calculated by determining the relative fluorescence units generated under steady state kinetics from which values of caspase-independent protease activity in the presence of the corresponding inhibitor was subtracted. Actinomycin D and TNF*α* were used as positive controls.

### Animal experiments

All animal experiments were in accordance with Swiss federal animal regulations and approved by the cantonal veterinary office of Zurich. Specific pathogen-free Balb/c mice 10–12 weeks of age (Harlan, Netherlands), syngeneic with the CT-26 colon carcinoma cell line, were kept on a 12 h day/night cycle with free access to food and water. Animal health, weight, and food intake were monitored daily, and animals were killed according to predefined criteria (signs of pain, reduction of food intake >50%, weight loss >20%). For subcutaneous tumour cell inoculations, CT-26 cells, cultured in the exponential growth phase, were treated with trypsin and washed in PBS. Cells were then suspended in serum-free medium, and 200 *μ*l (corresponding to 5 × 10^5^ cells) were injected subcutaneously. Therapy commenced after a subcutaneous tumour became detectable, which occurred after 4–5 days. Tumour growth was monitored daily with a sliding calliper, and tumour volume was calculated according to the ellipsoid formula 4/3 *π* × l/2 × w/2 × w/2, where l is the length and w is the width. Vehicles for intraperitoneal injection of LCL-30 and doxorubicin were 30% Cremophore (Sigma-Aldrich) and NaCl respectively. All animals received an equal number of intraperitoneal injections with active compound or appropriate vehicle controls (daily injections of LCL-30 and weekly injections of doxorubicin). Blood cell counts were determined with a Coulter AcT Diff counter (Beckman Coulter, Nyon, Switzerland). Plasma aspartate aminotransferase (AST), alkaline phosphatase, and creatinine were determined with the serum multiple analyzer (Ektachem DTSCII, Johnson & Johnson Inc., Rochester, USA).

### Histology and immunohistochemistry

Formalin-fixed tissue was paraffin-embedded, sectioned, and stained with H&E using standard techniques. For immunohistochemistry, tissue sections were incubated with anti-Ki-67 antibody (NeoMarkers). Pretreatment of sections, antibody incubation, and detection of primary antibody (Ventana DAB iView Kit) were performed on a Nexes immunohistochemistry staining system (Ventana Medical Systems, Tucson, AZ, USA). For CD31 staining, detection of primary antibody was performed with a Histofine staining kit (Nichirei Corporation, Tokyo, Japan) and diaminobenzidine (DAB) as a chromogen. All immunostains were counterstained with hematoxylin. TUNEL staining was performed with the *in situ* cell death detection kit (Roche Applied Science, Rotkreuz, Switzerland) according to the manufacturer's instructions. Ki-67 staining was quantified on 10 images with the analySIS^D imaging software using a semi-automatic thresholding algorithm (Olympus, Volketswil, Switzerland). Microvascular density was counted on 10 high-power fields of CD31-immunostains.

### Enzyme-linked immunosorbent assay (ELISA) for TNF*α*

TNF-*α* levels in plasma were determined by ELISA (Quantikine mouse TNF-*α*, R&D systems, Minneapolis, USA) following the manufacturer’s instructions. The lower detection limit of this assay is 5.1 pg ml^−1^.

### Quantitative real-time polymerase chain reaction

Total RNA was extracted from cells or tissue using TRIzol reagent (Invitrogen) following the manufacturer's instructions. Five micrograms of RNA were reverse transcribed to cDNA using the ThermoScript RT-PCR System (Invitrogen) kit. Quantitative real-time PCR amplification and data analysis were performed using an ABI Prism 7000 Sequence Detector System (PE Applied Biosystems, Rotkreuz, Switzerland). TaqMan gene expression assays (PE Applied Biosystems) were used to quantify mRNA expression of the respective genes. Results were quantified as fold induction in comparison to baseline after normalisation to 18S RNA (TaqMan ribosomal RNA control reagents, PE Applied Biosystems).

### Detection of LCL-30 and of endogenous sphingolipids

Liquid chromatography and mass spectrometry (LC-MS) analysis was performed in the Lipidomics Core of the Medical University of South Carolina on a Thermo Finnigan TSQ 7000 triple quadrupole mass spectrometer operating in a Multiple Reaction Monitoring (MRM) positive ionisation mode. Briefly, cell lysates or tumour homogenate fractions were fortified with internal standards for quantification, and lipids were extracted and analysed as previously described (Bielawski *et al*, 2006).

### Statistical analysis

Data represent mean±s.d. of *n* independent experiments. Mann–Whitney *U*-test or one-way ANOVA with Student–Newman–Keuls *post hoc* testing was used as appropriate to compare groups, using SPSS 12.0 (SPSS Inc., Chicago, USA). A *P*-value below 0.05 was considered to indicate statistical significance.

## RESULTS

### LCL-30 elicits cytotoxicity *in vitro*

**Figure 1:**
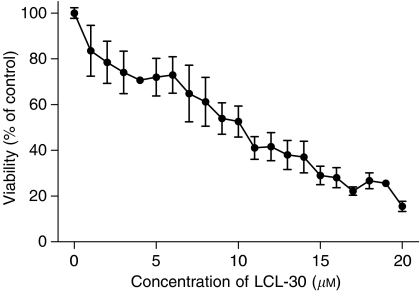
Cytotoxicity of LCL-30. CT-26 cells were incubated for 24 h with increasing concentrations of LCL-30, and viability was assessed by the MTT assay (IC50=10.6 *μ*M).

We previously tested the cytotoxicity of LCL-30 on a range of human and murine cancer cell lines (Dindo *et al*, 2006). For the present study, we focused on the colon cancer cell line CT-26, which can be used as a syngeneic *in vivo* model of colorectal cancer in Balb/c mice. LCL-30 treatment of CT-26 cells was able to effectively induce cell death *in vitro* in a dose-dependent manner (Figure 1). A 50% inhibition of cell viability (IC50) was achieved at 10.6 *μ*M. Time course experiments with different concentrations showed a time-dependent reduction of cell viability with a steady slope (not shown). These results were confirmed by trypan blue exclusion (not shown).

### LCL-30 targets mitochondria

**Figure 2:**
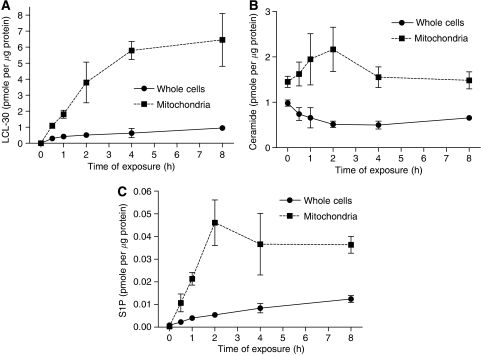
Cellular uptake of LCL-30 (**A**) and the resulting changes in endogenous ceramide (**B**) and sphingosine-1-phosphate (**C**) CT-26 were exposed for the indicated time to 10 *μ*M of LCL-30 and then separately analyzed by mass spectrometry for whole cells (squares) and mitochondrially enriched fractions (circles). Results are mean±s.d. of *n*=3 and normalised to protein content.

Ceramide has been detected in mitochondria (Dindo *et al*, 2006) as well as some ceramide-metabolising enzymes such as ceramide synthase and ceramidase. LCL-30 represents a cationic lipid designed to be enriched in the positively charged mitochondria. To investigate whether this mitochondrial accumulation occurs in CT-26, cells were treated with the IC50 concentration of LCL-30 (10 *μ*M) for up to 8 h. Whole cells and mitochondrially enriched fractions were isolated at different time points and subjected to mass spectrometry, allowing a detailed detection of endogenous sphingolipids as well as LCL-30. As illustrated in Figure 2A, LCL-30 was progressively taken up into cells with levels achieving approximately 0.95 pmole *μ*g^−1^ protein after 4 h of incubation. Notably, LCL-30 was significantly enriched in the mitochondrial fraction such that its levels in isolated mitochondria reached about 6 pmole *μ*g^−1^ protein by 4 h. Cellular and mitochondrial uptake appeared to level off after 4 h. These results demonstrate significant enrichment of LCL-30 in the mitochondria of CT-26 cells.

### LCL-30 induces an endogenous sphingolipid response

Next, the effects of LCL-30 on endogenous sphingolipids were examined. Cellular levels of total ceramide decreased gradually in response to LCL-30 treatment (Figure 2B), whereas mitochondrial levels peaked after 2 h before returning to normal. Levels of sphingosine and dihydrosphingosine reacted in an analogous fashion (not shown). Interestingly, cellular and mitochondrial levels of sphingosine-1-phosphate (S1P) rose continuously from almost undetectable levels (Figure 2C), with a more marked rise in the mitochondrially enriched fraction. These experiments demonstrate that LCL-30 enters CT-26 cells, is enriched in the mitochondrial fraction, and leads to a transient rise of endogenous mitochondrial ceramides as well as a marked rise of mitochondrial S1P.

### LCL-30 does not regulate ceramide-metabolising enzymes at the transcriptional level

The changes in cellular sphingolipid levels in response to treatment, especially the rapid rise of S1P levels, prompted us to analyse the expression levels of key enzymes in the metabolic conversion of ceramide to sphingosine and further to sphingosine-1-phosphate. Quantitative real-time PCR was performed for acid ceramidase (Asah1), neutral ceramidase (Asah2), alkaline ceramidase (Asah3), sphingosine kinase 1 (SphK1), and sphingosine kinase 2 (SphK2). Neutral ceramidase was never detectable. None of the expressed enzymes showed any changes in their transcript levels during 8 h of treatment with LCL-30 (data not shown).

### LCL-30 decreases cellular ATP levels

**Figure 3:**
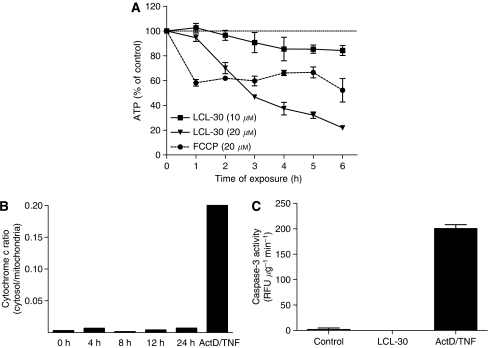
(**A**) Cellular ATP content during exposure to 20 *μ*M FCCP (circles), 10 *μ*M LCL-30 (squares) and 20 *μ*M LCL-30 (triangles). Results are mean±s.d. of *n*=4 and normalised to protein content. (**B**) Ratio of cytosolic to mitochondrial cytochrome *c* at different durations of incubation with 10 *μ*M LCL-30, as assessed by ELISA. (**C**) Caspase-3-activity after 6 h of exposure to 10 *μ*M LCL-30.

As the above experiments showed LCL-30 to be enriched in mitochondria, we assessed whether mitochondrial function was affected. Cellular ATP levels showed a continuous time- and dose-dependent decrease, with different kinetics than the mitochondrial uncoupler FCCP (Figure 3A). Surprisingly, we could not detect cytosolic cytochrome *c* release by ELISA after 8 or 24 h of incubation with LCL-30 at a concentration of 10 *μ*M (Figure 3B). In line with this observation, we could not detect any caspase 3 (Figure 3C) or caspase 8 activity (data not shown). Actinomycin D and TNF*α* (ActD/TNF) were used as positive controls for these assays. Taken together, LCL-30 accumulates in mitochondria of CT-26 cells and decreases ATP production, ultimately causing cell death, without detectable cytochrome *c* release or caspase activation.

### Dose-finding and toxicity in mice

A major aim of this study was to investigate the effects of the long chain pyridinium ceramide LCL-30 in an animal model. To define the toxicity and the tolerable dose, escalating doses of LCL-30 dissolved in 30% cremophore were administered to mice intraperitoneally. A single dose of 100 mg kg^−1^ was lethal, while 50 mg kg^−1^ was lethal after several doses. Toxicity manifested itself by an eosinophilic inflammatory reaction of the peritoneum, followed by fibrinous exudation and fibrous organisation, resulting in severe intraperitoneal adhesions and ileus. No other toxic effects were identified either by histological analysis of internal organs, including brain and bone marrow, or by blood counts and plasma tests (aspartate aminotransferase, alkaline phosphatase, and creatinine). Daily injections of 20 mg kg^−1^ for 1 week were tolerated. This dose was then combined with different doses of doxorubicin. The addition of 6 mg kg^−1^ doxorubicin to 20 mg kg^−1^ LCL-30 was also established as safe. Average weight loss in animals undergoing treatment was 6.8% (control), 9.1% (doxorubicin), 12.7% (LCL-30), and 13.9% (LCL-30+doxorubicin). None of these animals had to be killed according to the pre-defined criteria. Thus, the *in vivo* application of LCL-30 caused localised inflammation and did not result in systemic toxicity.

### Pharmacokinetics of LCL-30

**Figure 4:**
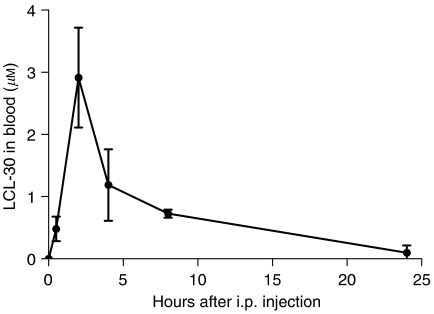
Concentration of LCL-30 in blood after a single intraperitoneal injection of 20 mg kg^−1^. Results are mean±s.d. of *n*=4 and normalised to protein content.

After defining the tolerable dose of LCL-30 *in vivo*, we sought to identify the pharmacokinetic behaviour of this compound. The levels of LCL-30 as well as endogenous sphingolipids were determined by mass spectrometry after a single intraperitoneal dose of 20 mg kg^−1^ of LCL-30 (Figure 4). LCL-30 was rapidly absorbed from the peritoneal cavity, leading to a peak blood concentration after 2 h. Elimination from blood was almost complete after 24 h. Concentrations were determined separately for plasma and the cellular components of blood: at its peak level, LCL-30 partitioned equally into the aqueous as well as the cellular phase of blood, with slower elimination from the cellular compartment.

### Therapeutic efficacy of LCL-30 in subcutaneous tumour grafts

**Figure 5:**
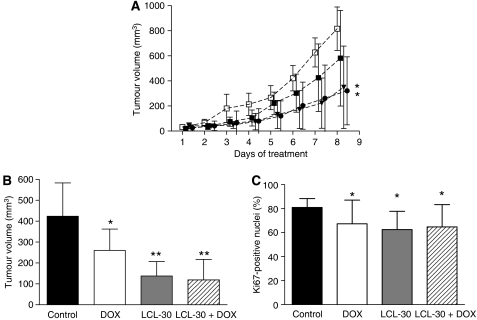
(**A**) Progression of subcutaneous tumours in animals receiving daily injections with vehicle (open squares), doxorubicin (closed squares), LCL-30 (triangles), or LCL-30+doxorubicin (circles), ^*^P=0.027 *vs* control (ANOVA); (**B**) Volume of explanted tumours after the end of treatment, ^*^P<0.05, ^**^P<0.01 *vs* control (ANOVA with Student–Newman-Keuls *post hoc* test). (**C**) Proliferative activity of treated tumours assessed by Ki67 immunohistochemistry, ^*^P<0.001 *vs* control (ANOVA with Student–Newman-Keuls *post hoc* test). Results are mean±s.d. of *n*=8.

The next step was the assessment of the therapeutic efficacy of LCL-30 in the treatment of established subcutaneous tumour grafts. Pharmacokinetic data led us to choose a dosing regimen of once per day for LCL-30. After the establishment of solid tumours, animals were randomised to one of four treatment regimens and received an equal number of injections over the course of 1 week. At the beginning of treatment, there were no differences between the animal groups. The growth curves under treatment are shown in Figure 5A; whereas LCL-30 significantly reduced tumour growth, doxorubicin did not cause a significant growth reduction. The combination of LCL-30 and doxorubicin did not add to the efficacy of LCL-30 alone. Figure 5B shows the volumes of explanted tumours measured ex-vivo, where doxorubicin-treated tumours were also significantly smaller than controls. Again, LCL-30 was more efficacious than doxorubicin, and the combined treatment did not produce any additive effects. It should be noted that the absolute values of tumour volumes in this protocol are lower due to the systematic overestimation of tumours measured through the skin of live animals. Importantly, the anti-tumour effect could not be explained by an unspecific systemic inflammation, as plasma levels of TNF*α* remained undetectable in all animals, while positive controls (LPS injection) yielded values of 1098±261 pg ml^−1^ (not shown). Therefore, LCL-30 demonstrates significant efficacy against colon cancer in this *in vivo* model.

### Histological assessment of subcutaneous tumours

Histological assessment of H&E-stained tumour sections revealed no differences between the groups with respect to tumour morphology or necrotic area. To analyse whether apoptotic cell death plays an important role in tumours, we examined TUNEL stains. There was a low number of single-cell apoptosis in all groups with no differences between groups (not shown). However, the mitotic count was reduced from a baseline of six per high power field (HPF) to five in the doxorubicin group and to four in the LCL-30 and LCL-30+doxorubicin groups. This led us to use Ki67-immunostaining to further analyse the proliferating tumour cell fraction. The results also showed a reduced proliferative activity of treated tumours (Figure 5C). It is noteworthy that microvascular density (as assessed by CD31-immunostaining) was not different between groups (not shown).

### Sphingolipid profiles of subcutaneous tumours

**Figure 6:**
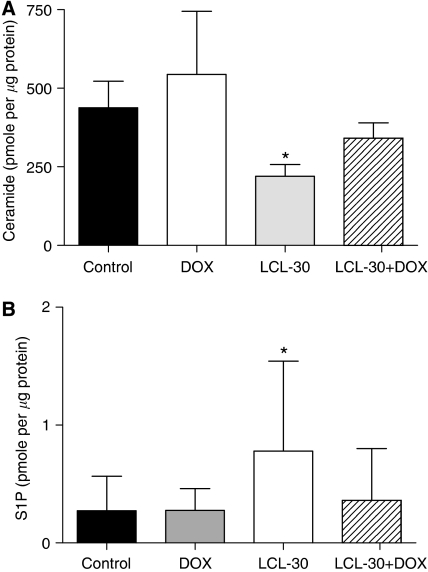
(**A**) Levels of endogenous ceramide in tumours after 1 week of treatment with the indicated regimen, ^*^P=0.098 *vs* control. (**B**) Levels of sphingosine-1-phosphate in tumours after 1 week of treatment with the indicated regimen, ^*^P=0.045. Results are mean±s.d. of *n*=4 and normalised to protein content.

After completion of treatment, tumour samples were also subjected to mass spectrometry analysis of sphingolipid content. Importantly, 24 h after the last injection of a 1-week treatment course, LCL-30 was detected at 0.22 (±0.09) and 0.23 (±0.08) pmole *μ*g^−1^ protein in the LCL-30 and LCL-30+doxorubicin-treated groups, respectively. The content of endogenous ceramides decreased in the LCL-30-treated tumours, an effect that was less pronounced after doxorubicin co-treatment (Figure 6A). Similar to the *in vitro* effects, LCL-30 caused an increase in S1P levels (Figure 6B), while sphingosine levels were lowered by LCL-30 treatment (not shown). Thus, LCL-30 appears to concentrate on tumours even when it has been cleared from the blood with persistent effects on tumour sphingolipids that recapitulate its effects in tissue culture.

## DISCUSSION

This study is the *in vivo* continuation of our previous experiments with LCL-30, the cationic water-soluble analogue of C_16_-ceramide, in which we could demonstrate that LCL-30 accumulates in the mitochondria of SW403 colorectal cancer cells and induces mitochondrial swelling, cytochrome *c* release, caspase activation, and eventually cell death (Dindo *et al*, 2006). Here, we expand our analysis to the murine colon carcinoma cell line CT-26 cells and use CT-26 cells in syngeneic Balb/c mice as an *in vivo* model of colorectal cancer.

LCL-30 was cytotoxic for CT-26 cells in a dose- and time-dependent fashion, in analogy to other cell lines previously tested (Dindo *et al*, 2006). Cellular fractionation and mass spectrometric analyses showed LCL-30 to be enriched in the mitochondrial fraction, in line with published data on cationic *ω*-pyridinium analogues of C_6_ and C_16_ ceramide (Novgorodov *et al*, 2005; Dindo *et al*, 2006; Senkal *et al*, 2006). Incubation with LCL-30 led to a dose- and time-dependent decrease of cellular ATP-levels, pointing to a breakdown of mitochondrial respiration, as already described for the *ω*-pyridinium C_6_ analogue LCL-29 (Novgorodov *et al*, 2005). Yet to our surprise, hallmarks of mitochondrially mediated apoptotic cell death, such as cytochrome *c* release or caspase activation, could not be detected. The mechanism of LCL-30-mediated cell death in CT-26 remains unclear, although ceramide has been implicated as an endogenous mediator of caspase-independent programmed cell death (Thon *et al*, 2005). Delineating the differences between cell lines that show caspase activation (SW403) and those without (CT-26) might help define the different mechanisms involved.

Exposure to LCL-30 led to a transient depression of whole-cell ceramide levels, whereas mitochondrial ceramide levels showed a transient rise. This ceramide response is somewhat different from SW403, which show a mitochondrial decrease of ceramide levels (Dindo *et al*, 2006). Importantly, the rapid and pronounced rise of mitochondrial S1P levels is comparable between both cell lines, raising the possibility of the presence of a mitochondrial sphingosine kinase (SphK). Additional experiments with isomers of LCL-30 have revealed S1P to be derived from endogenous sources and not from the breakdown of LCL-30 (Bielawska A, unpublished). S1P has been primarily regarded as a counter player of ceramide activity, although the intracellular compartmentalisation and the biological context (Ikeda *et al*, 2003) are important for its biological effects. Future experiments should take intracellular distribution of sphingosine kinase proteins into account. While the activation of SphK1 leads to reduced apoptosis and improved proliferation, activation of SphK2 has been associated with enhanced cell death (Liu *et al*, 2003), which has been attributed to differential localisation in ER *vs* cytosol (Wattenberg *et al*, 2006). Neither SphK1 nor SphK2 has been detected in mitochondria. Nevertheless, there is evidence for additional sphingosine kinase activity (Fukuda *et al*, 2003), which might be responsible for the rise in mitochondrial levels of S1P in response to LCL-30. Alternatively, enhanced levels of S1P might be explained by an inhibition of S1P-degrading enzymes S1P Lyase or S1P phosphatase by LCL-30 (Oskouian *et al*, 2006). At present, the exact role of S1P produced in response to exogenous treatment with a ceramide analogue remains elusive: it could be an antiapoptotic escape mechanism, a cytotoxic signal, or an epiphenomenon.

Another focus of this study was to assess the safety and efficacy of LCL-30 in an *in vivo* mouse tumour model. The maximum tolerable dose could be established in dose-escalation studies. Interestingly, dose-limiting toxicity manifested itself as a local peritoneal reaction. The lack of organ-specific toxic effects is encouraging as it carries two important implications. First, it hints at a certain degree of tumour-selectivity of LCL-30, and second, the locally toxic effects of LCL-30 might be circumvented by alternative modes of application.

After a single intraperitoneal injection, LCL-30 reached a peak concentration in blood within 2 h and was cleared within 24 h. Peak concentrations were lower than LC50 *in vitro*, although a higher peak between the first two pharmacokinetic sampling points (30 min and 2 h) cannot be excluded. Clearance was somewhat slower than for C_6_ pyridinium, which was already cleared from the circulation after 4 h by renal excretion (Senkal *et al*, 2006), suggesting that ceramides with longer acyl chains might be cleared from the circulation more slowly. Such pharmacokinetic behaviour might be beneficial for therapeutic purposes.

Treatment of established subcutaneous tumours over the course of 1 week showed LCL-30 to be an efficacious compound *in vivo*. Cytotoxic effects on tumours *in vivo* were less than expected from *in vitro* experiments, possibly due to insufficient peak concentrations being reached *in vivo*. Inhibition of tumour proliferation might have been caused by an unspecific inflammatory response to peritoneal injection of a peritoneal irritant. As TNF*α* could not be detected in the plasma of any animal, this is highly unlikely. Relative ceramide levels were much higher in solid tumours than in cell culture, which might be caused by a different sphingolipid composition of tumour cells growing *in vivo*. Solid tumours also contain additional cell types, such as stromal or infiltrating blood-derived cells, with a high ceramide content (Dahm *et al*, 2006). On the basis of *in vitro* data showing synergistic cytotoxicity of doxorubicin with LCL-30 (Dindo *et al*, 2006), doxorubicin was also tested alone and in combination treatment. In contrast to the *in vitro* observation, doxorubicin conveyed no additive effect compared to LCL-30 alone. This might be related to the dosing schedule where LCL-30 was administered daily and doxorubicin, once per week. Weekly administration of doxorubicin was based on established dosing regimens (Yoneda *et al*, 1999; Dubois *et al*, 2002).

In summary, we present the first *in vivo* application of a long-chain cationic ceramide for the treatment of experimental metastatic colorectal cancer, together with its pharmacokinetic parameters. Although cytotoxic for CT-26, the mechanism of cell death was different from the previously studied SW403, and doxorubicin did not convey additive effects *in vivo*. Nevertheless, LCL-30 was an efficacious and safe agent. Future studies should further elaborate on the mechanism of cell death and aim to identify an alternative application route as well as more effective partners for combination treatment.
